# Exogenous Salicylic Acid Modulates the Response to Combined Salinity-Temperature Stress in Pepper Plants (*Capsicum annuum* L. var. Tamarin)

**DOI:** 10.3390/plants9121790

**Published:** 2020-12-17

**Authors:** Ginés Otálora, María Carmen Piñero, Jacinta Collado-González, Josefa López-Marín, Francisco M. del Amor

**Affiliations:** Department of Crop Production and Agri-Technology, Murcia Institute of Agri-Food Research and Development (IMIDA), C/Mayor s/n, 30150 Murcia, Spain; mariac.pinero2@carm.es (M.C.P.); jacinta.collado@carm.es (J.C.-G.); josefa.lopez38@carm.es (J.L.-M.)

**Keywords:** heat shock stress, salinity, combined stress, NaCl, temperature, extreme weather

## Abstract

Growers in the cultivated areas where the climate change threatens the agricultural productivity and livelihoods are aware that the current constraints for good quality water are being worsened by heatwaves. We studied the combination of salinity (60 mM NaCl) and heat shock stress (43 °C) in pepper plants (*Capsicum annuum* L. var. Tamarin) since this can affect physiological and biochemical processes distinctly when compared to separate effects. Moreover, the exogenous application of 0.5 mM salicylic acid (SA) was studied to determine its impacts and the SA-mediated processes that confer tolerance of the combined or stand-alone stresses. Plant growth, leaf Cl^−^ and NO_3_^−^ concentrations, carbohydrates, and polyamines were analyzed. Our results show that both salinity stress (SS) and heat stress (HS) reduced plant fresh weight, and SA only increased it for HS, with no effect for the combined stress (CS). While SA increased the concentration of Cl^−^ for SS or CS, it had no effect on NO_3_^−^. The carbohydrates concentrations were, in general, increased by HS, and were decreased by CS, and for glucose and fructose, by SA. Additionally, when CS was imposed, SA significantly increased the spermine and spermidine concentrations. Thus, SA did not always alleviate the CS and the plant response to CS cannot be directly attributed to the full or partial sum of the individual responses to each stress.

## 1. Introduction

Global warming is increasing the number, intensity, and duration of abiotic stress combinations worldwide, which impair crop growth, yield, and product quality [1]. The plant response to such combinations of stresses is complex, involving a multitude of molecular signaling pathways that control several responses, which, in turn, may interact with one another [2]. Therefore, it has been suggested that each abiotic stress combination requires new research as it should be studied as an entirely new stress [3]. Increases in both air temperature and the salinity of irrigation waters will be two of the major constraints to human food production in the coming years. Therefore, improved agronomic management is of paramount importance to develop key adaptation strategies intended to increase stress tolerance in crops [4]. Salinity is a serious concern, being an increasing problem in agriculture because of the competition of good-quality water from industry and the progressive salinization of aquifers and other water resources, especially in arid and semiarid regions. This scarcity of good-quality water is dramatically accentuated by the rainfall alterations provoked by climate change. In addition, extreme temperatures associated with prolonged heatwaves impact more than 10% of land surfaces [5]. A cautionary example is that of the summer of 2003, when a heatwave that impacted Europe resulted in a 30% reduction in ecosystem gross primary production [6], while a more devastating heatwave during 2010 in Russia resulted in an estimated 50% reduction in gross primary production [7].

Plant growth regulators (phytohormones) play an essential role in plant responses to biotic and abiotic stress [8,9]. Salicylic acid (SA) is an endogenous plant hormone and different studies have pointed out the positive effects of using this hormone as a treatment to stimulate plant growth under abiotic stress conditions, including combinations of drought, heat, and salinity [10,11], since SA acts as a key signaling molecule under such conditions. Its activity is essential for basal defense and systemic acquired resistance [12] and leads to the reprogramming of the expression of genes and the synthesis of proteins, affecting a number of metabolic processes [13]. However, the exact mode of action of SA is still poorly understood, as SA signaling is not a simple linear route and SA may interact with several other stress-related compounds [13].

Pepper (*Capsicum annuum* L.) is a salt-sensitive crop [14], and the use of poor-quality waters causes a significant reduction in yield, especially in the marketable yield [15]. Thus, pepper yield and quality are affected by temperature and salinity stresses [16], with the optimal temperature range for growth being 20–30 °C, while above 32 °C pepper shows pollination and fertilization problems and a significant reduction in fruit quality [17]. However, vegetative and generative growth can be differentially affected [18], with specific heat-stress-related genes involved in determining heat tolerance [19]. Pepper is an important horticultural crop due to its economic and nutraceutical values and is cultivated worldwide, especially in greenhouses of the Mediterranean-climate areas. To our knowledge, this is the first paper addressing combined drought and salinity stress in pepper, with the addition of SA. The purpose of this research was to gain insights into the combined effect of salinity and drought in pepper, and its response to exogenous (leaf-sprayed) SA. Thus, the specific objectives were: (i) to examine the extent to which the studied stresses had univocal effects and the degree of interaction when plants were submitted to both stresses, (ii) to evaluate growth and ion-specific responses, to characterize the sum of the stresses, (iii) to determine whether the combined stresses had a synergic or antagonist effect regarding the leaf carbohydrate concentration, (iv) to reveal whether polyamines modulated the general response, and (v) to determine whether the spraying of SA onto plants subjected to the combination of stresses, or the individual stresses, altered the response of the studied parameters, with mitigation of the effects of the stresses.

## 2. Results

### 2.1. Plant Growth

**Figure 1:**
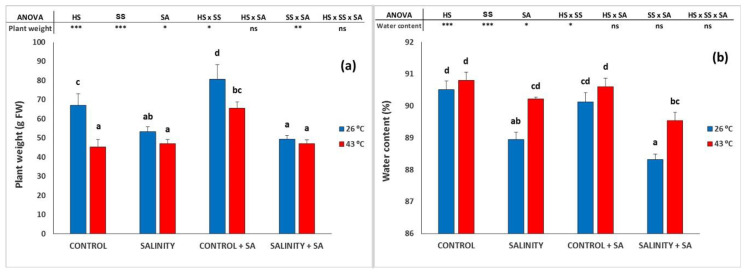
Effect of heat-shock and salinity, with or without salicylic acid, on the fresh weight of the aerial part (leaves and stems) of pepper plants (**a**), and on the leaf water content (**b**) in the leaves of pepper plants. The * refers to significant differences at the level of *p* ≤ 0.05; ** *p* ≤ 0.005; *** *p* ≤ 0.001; n.s., not significant. Different lowercase letters denote significant differences between columns, *p* < 0.05 HS is heat stress; SS is salinity stress; SA is salicylic acid.

Plant fresh weight (aerial part) decreased with the exposure to salinity and heat (Figure 1a). Thus, the fresh weight values recorded for control plants were reduced by 20.4% and 32.4% when salinity or heat stress, respectively, was imposed, but salinity did not reduce the growth of plants already exposed to heat stress (combined stress). The spraying of SA boosted the fresh weight of non-stressed plants (20.3%) and of heat-stressed plants (39.6%); however, this effect was clearly diminished with salinity alone or with the combined stress.

The leaf water content (Figure 1b) was reduced by salinity, especially when no HS was imposed; thus, HS increased the water content of those leaves submitted to SS. Additionally, SA did not affect the response to HS or SS alone or to the combination of both stresses. Thus, in our controlled conditions, with no restrictions in the water supply and a nutrient balance in the rhizosphere, heat increased the leaf water content in salinized plants.

### 2.2. Ion Concentrations

**Figure 2:**
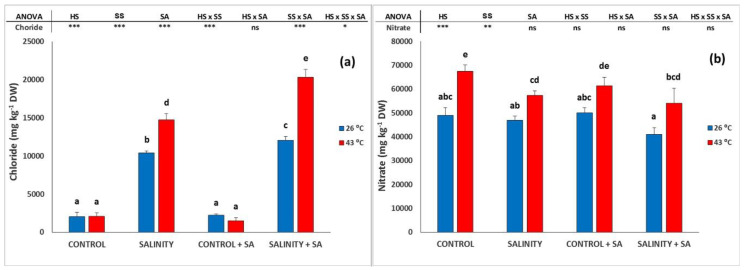
Effect of heat-shock and salinity, with or without salicylic acid, on the Cl^−^ (**a**) and NO_3_^−^ (**b**) concentrations in the leaves of pepper plants. The * refers to significant differences at the level of *p* ≤ 0.05; ** *p* ≤ 0.005; *** *p* ≤ 0.001; n.s., not significant. Different lowercase letters denote significant differences between columns, *p* < 0.05 HS is heat stress; SS is salinity stress; SA is salicylic acid.

As expected, the leaf Cl^−^ increased when Cl^−^ in the nutrient solution was increased (Figure 2a), and HS dramatically increased the Cl^−^ concentration in the leaves of SS plants. Surprisingly, we found a differential effect with respect to the application of SA; thus, in leaves sprayed with SA, the concentration of this ion significantly increased at both temperatures by 16.3% (26 °C) and 38.1% (43 °C). The concentration of nitrate in the leaves was increased by 37.6% by HS with respect to control plants, and HS increased nitrate by 22.1% when combined with SS. However, the application of SA had no effect on the nitrate concentration under HS or SS (Figure 2b).

### 2.3. Total Soluble Sugars

**Figure 3:**
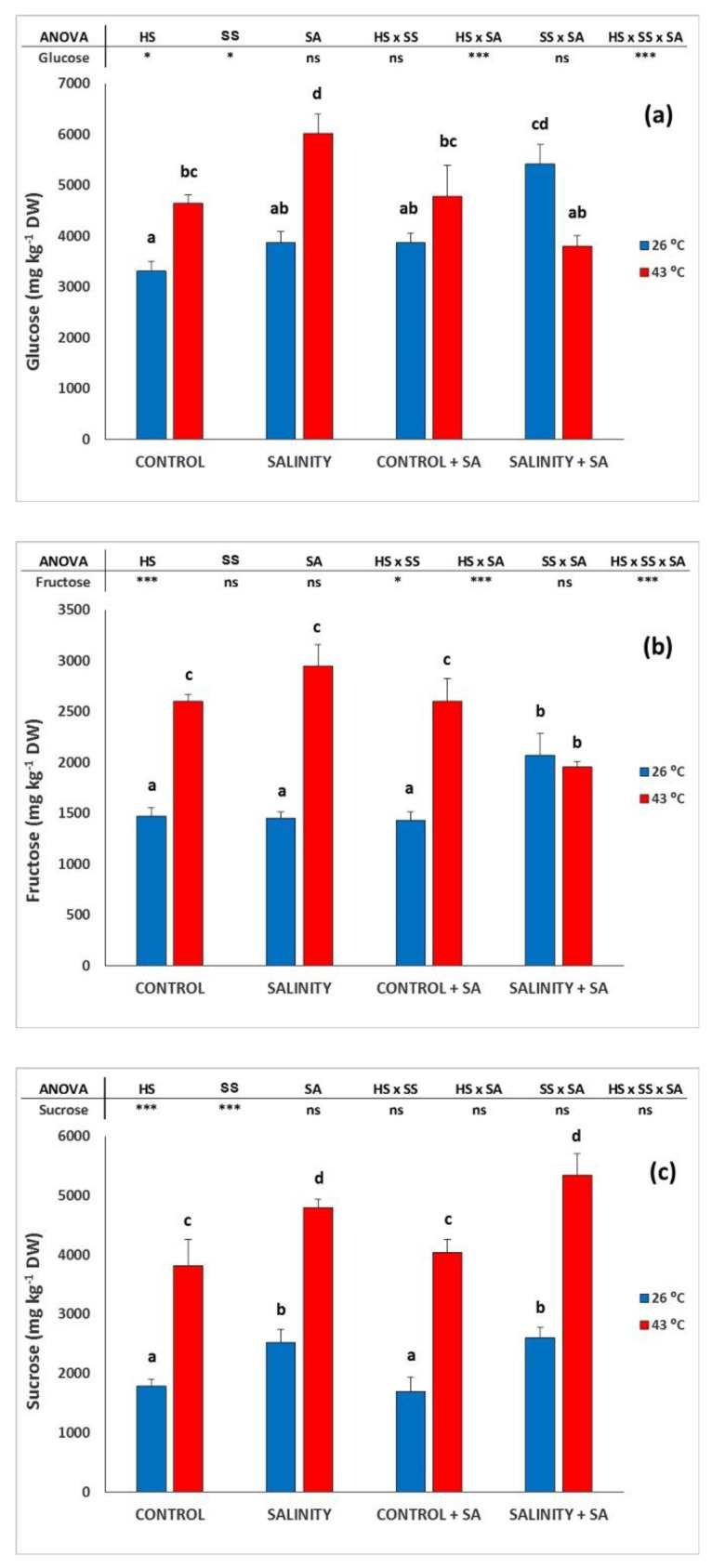
Effect of heat-shock and salinity, with or without salicylic acid, on the carbohydrates (glucose (**a**), fructose (**b**), and sucrose (**c**)) in the leaves of pepper plants. The * refers to significant differences at the level of *p* ≤ 0.05; *** *p* ≤ 0.001; n.s., not significant. Different lowercase letters denote significant differences between columns, *p* < 0.05 HS is heat stress; SS is salinity stress; SA is salicylic acid.

The carbohydrates concentrations in the leaves are presented in Figure 3. When HS was imposed on control plants, the glucose concentration (Figure 3a) was increased by 40.1%, but this effect of temperature was augmented by salinity, and with the CS, the glucose concentration increased by 55.75%. The SA application had a lower effect on non-salinized plants, but caused an increase at ambient temperature (SS plants), but not for HS. The patterns observed for the other carbohydrates measured (fructose (Figure 3b) and sucrose (Figure 3c)) were similar to those for glucose.

### 2.4. Polyamines Analysis

**Figure 4:**
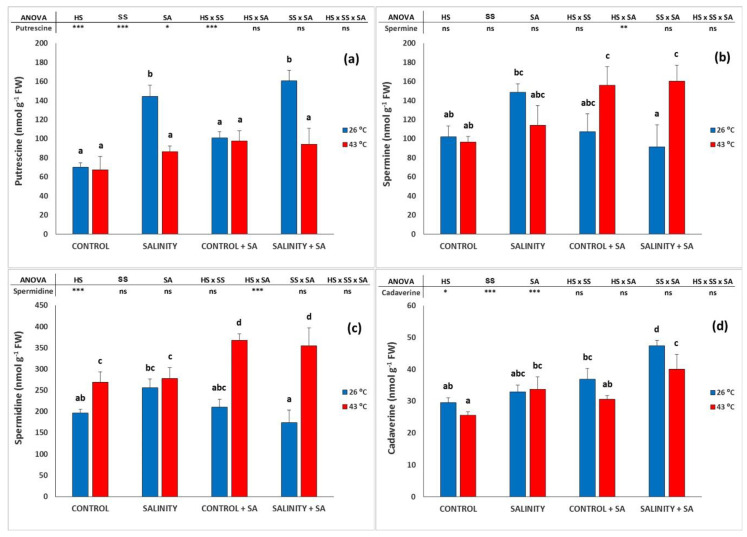
Effect of heat-shock and salinity, with or without salicylic acid, on the polyamines (putrescine (**a**), spermine (**b**), spermidine (**c**), and cadaverine (**d**)) in the leaves of pepper plants. The * refers to significant differences at the level of *p* ≤ 0.05; ** *p* ≤ 0.005; *** *p* ≤ 0.001; n.s., not significant. Different lowercase letters denote significant differences between columns, *p* < 0.05. HS is heat stress; SS is salinity stress; SA is salicylic acid.

The concentration of putrescine was not affected by HS (Figure 4a); however, when only SS was applied, we found a dramatic increase in this polyamine, from 70.2 nmol g^−1^ FW to 144.5 nmol g^−1^ FW. Interestingly, with the CS, its concentration in the leaves was lower at 86.6 nmol g^−1^ FW. The application of SA did not alter this response in the leaves, and only in SS plants did it produce a significant change. Spermine was not significantly altered by heat or salinity or by the combined stress when no SA was sprayed (Figure 4b), but SA applied to plants that were submitted to salinity or to both stresses jointly originated a significant increase that was not observed with no HS and without application of SA. In this way, spermine was increased by 61.5% and 40.4% when SA was applied under HS and the combined stress, respectively. Spermidine (Figure 4c) had a similar behavior with the exception that significant differences were observed for HS plants, and SA gave increases of 36.4% and 27.7% when applied to the HS and CS plants, respectively. Note that the combined stress did not significantly increase the spermidine concentration with respect to non-salinized plants. Cadaverine had an interesting and distinct pattern with respect to spermine or spermidine (Figure 4d): SA increased the concentration of cadaverine in the absence of stresses, and moreover, it produced a reduction in the response when plants were submitted to CS, as compared to SA application under SS alone.

### 2.5. Principal Component Analysis (PCA)

Principal component analysis (PCA) was applied to our results to see the relationship of the variables studied with the temperature and salinity conditions, together with the effect of SA.

**Figure 5:**
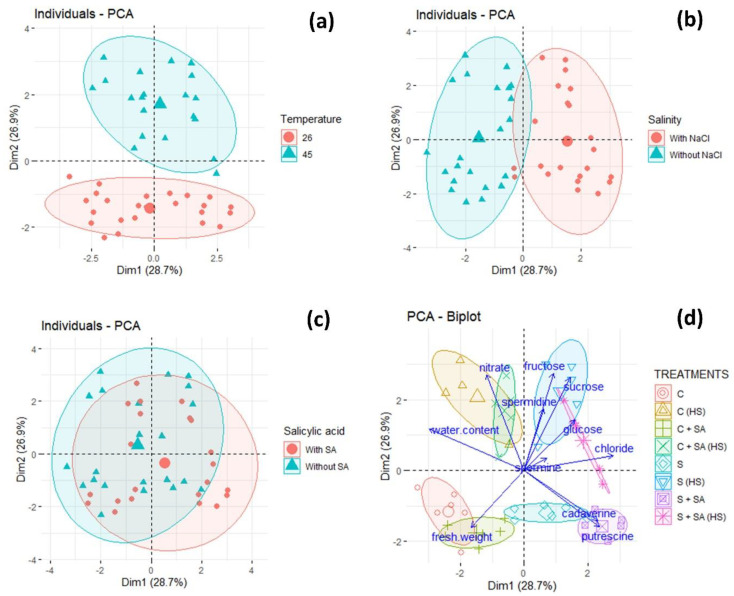
Principal component analysis (PCA) of the parameters analyzed in pepper plants cultivated with or without heat-shock and/or salinity, and with or without salicylic acid. (**a**) Cases are grouped by the temperature conditions applied; (**b**) cases are grouped by the salinity conditions applied; (**c**) cases are grouped by the salicylic acid conditions applied; and (**d**) PCA biplot where the cases are grouped by the treatments applied: C, control; S, salinity; SA, salicylic acid; HS, heat stress.

The first two main components explain 56% of the total variability of the 11 variables analyzed. The points represent the measurements and the arrows, whose length indicates the amount of variance explained by each variable, represent the variables. The abscissa axis “Dim1” is mainly a salinity gradient, where the variables located to the right of the axis are more affected by salinity than those located to the left (Figure 5b). In turn, the ordinate axis (Dim2) is a temperature gradient (Figure 5a). The highest temperature (heat stress) is at the top of the axis, while the lowest temperature is at the bottom of the axis.

Figure 5d shows how the glucose, fructose, sucrose, spermine, and spermidine variables are grouped by the heat and salinity stress conditions. Moreover, the cadaverine and putrescine variables are affected by salinity but not heat stress. The control treatment and heat stress mainly affect the water content and nitrate. Conversely, the fresh weight variable is grouped in the control treatment.

## 3. Discussion

When plants are submitted to an unfavorable combination of stresses, the exogenous application of phytohormones has been pointed out as a method to assist their adaptation to these conditions [11]. SA is a plant hormone that regulates growth and several biochemical and physiological processes in plants [20]. Our data show that SA had an effect on shoot FW under control and HS conditions, but not for the combined stress (HS and SS). Khan et al. [21] reported that application of 0.5 mM SA to non-stressed wheat plants improved the photosynthetic characteristics significantly, resulting in greater growth. Additionally, SA directly influences the activity of enzymes that participate in mechanisms of heat tolerance or induces the genes involved [22]. This agrees with our data showing that SA ameliorated HS, giving an increase in fresh weight under HS. However, for this pepper cultivar and intensity of SS (60 mM NaCl), no beneficial effect of SA was observed for fresh weight. Pancheva et al. [23] reported a decrease in the growth of leaves and roots and delayed leaf emergence of barley plants when SA (0.1 mM–1 mM) was applied exogenously. However, Gunes et al. [24] reported interesting results for maize about the exogenous application of SA (0.1 to 1 mM) and its effect on nutrient concentrations; SA increased plant growth significantly, in both saline and non-saline conditions. In contrast, Hayat et al. [25] underlined that SA could exert deleterious effects on plants under normal growth conditions, as a decline in net photosynthetic rate, and the transpiration rate was observed in maize plants. Therefore, in our work, for the chosen cultivar, SA acted beneficially when salinity was applied, rather than under the control or HS conditions. Barba-Espin et al. [26] reported that SA (100 µM) negatively affected the growth parameters of pea plants subjected to salt stress (70 mM NaCl). This was attributed to an SA-induced increase in oxidative damage during NaCl stress [27], together with a decrease in the NO_3_^−^ concentration; this latter effect of SA was not found in our study.

SA exacerbated the uptake of Cl^−^ by salinized plants at both ambient and extreme temperature. Intriguingly, some studies found that SA increased the root length by 45% compared to the control plants [28], an effect that could favor an increase in the uptake of this anion. Additionally, the observed effect was even bigger at higher temperatures, which would have induced a higher potential transpiration flux. Interestingly, Barba-Espín et al. [26] indicated that the percentage of leaves showing chlorosis symptoms was increased after application of SA under SS. However, in our experiment, the leaves did not have necrotic margins despite the fact that the leaf Cl^−^ concentration was boosted by the application of SA under SS. Moreover, the increase in Cl^−^ and these effects of SA on growth and ion concentrations may depend on the plant species [24]. In our study, N assimilation was not enhanced by SA, in contrast to the findings of Nazar et al. [29] and Khan et al. [21] for salinity (for mungbean and wheat, respectively), but HS caused an increase in the accumulation of this nutrient for our pepper cultivar. Hayat et al. [25] indicated that the treatment of maize plants with lower concentrations of SA enhanced N uptake and the activity of the enzyme NR, whereas higher concentrations were inhibitory (they did not lower Cl^−^ accumulation or raise the N concentration). Thus, in that crop, SA likely acts differentially depending on the concentration sprayed. It should be underlined that the accumulation of this toxic anion (Cl^−^) in the leaves could have impaired the photosynthetic machinery, leading to the reduction in growth observed for CS, but clearly, the response of growth to that stress combination is qualitatively different to the sum of the individual stress responses. Silva et al. [30] found that the combined stress (HS and SS) was more harmful to *Jatropha curcas* than the stresses applied individually, with a main role for the detrimental effect of Cl^−^ (enhanced uptake of this toxic ion), whereas in tomato such interaction produced a higher degree of tolerance [31]. However, the work of Silva et al. [30] and ours were performed at significantly higher temperatures.

Sugars are a major source of carbon and energy in plants, but also play an important signaling role in many physiological processes [32]. Recent works have shown that, under abiotic stress, sucrose import is blocked, and this can have a critical effect on generative plant growth, causing the abortion of fruits [33]. Our data for the leaves indicate that the sugar contents were, in general, enhanced by the HS imposed, but SA had an effect in the plants subjected to the CS, decreasing glucose and fructose but not sucrose. Elwan et al. [34] observed that spraying pepper plants with a low concentration of SA (1 µM) decreased significantly the sugars content of leaves but increased it in fruits, and attributed this to the role of SA in energy status balancing, translocation, and storage of assimilates. The research of Zhou et al. [35] agrees with our data in that heat stress significantly increased the glucose, fructose, and sucrose in the leaves of tomato. In general, it is stated that high temperatures can cause a rapid consumption of carbohydrates for the maintenance of respiration [36], but we can envisage that, in our conditions, the photosynthetic metabolism was increased and able to counteract the demand for carbohydrates of this cultivar. Thus, Zhang et al. [32] indicated that plants supplemented with SA under HS showed a significant enhancement in the content of photosynthetic pigments. Carbohydrates have a fundamental role in cell osmotic adjustment and in the maintenance of membrane integrity [37], and an increase in the total soluble sugars was observed in heat-tolerant genotypes [19], whilst for sensitive cultivars, HS has been associated with disturbed carbohydrate metabolism due to an increase in oxidative damage [38].

Polyamines have an important role in the regulation of the plant response to abiotic stresses [39,40,41]. Recently, Tajti et al. [42] identified, in Arabidopsis SA-deficient mutants, a possible crosstalk between the SA and polyamine signaling pathways, whilst Collado-González et al. [43] reported in cauliflower that extreme heat increased the content of all polyamines. Gupta et al. [44] indicated that the principal enzymes involved in the polyamine biosynthesis pathway are under complex metabolic and developmental control. Tanou et al. [45] reported that polyamines ameliorated NaCl stress in five-month-old sour orange, but in our recent work in melon heat stress provoked a significant effect on the polyamines response [46], whilst Upadhyay et al. [47] indicated in tomato leaves that heat stress (42 °C) led to a decrease in the levels of free putrescine. As indicated previously, we did not find a general summative response to the combined stress or a general amelioration effect of the SA against the stresses applied jointly or individually. Such a combination of stresses should be studied as a new state of abiotic stress that demands new, complex acclimation responses [48] in which SA may also trigger new signaling pathways.

## 4. Materials and Methods

### 4.1. Growth Conditions and Treatments Applied

Pepper plants (*Capsicum annuum* L.) var. Tamarin (Enza Zaden España S.L., Almeria, Spain), which are bloky pepper type, were pregerminated in commercial seed trays (El Jimenado S.A., Torre-Pacheco, Murcia). Seedlings were transplanted into 40 5-L pots filled with coconut fiber and watered with a modified Hoagland solution. They were grown in a climate chamber with the characteristics described by del Amor et al. (2010), with fully controlled environmental conditions. Photosynthetically active radiation (PAR) of 250 µmol m^−2^ s^−1^ was provided by a combination of fluorescent lamps (TL-D Master reflex 830 and 840, Koninklijke Philips Electronics N.V., Amsterdam, The Netherlands) and high pressure lamps (Son-T Agro, Koninklijke Philips Electronics N.V., The Netherlands). The initial day/night conditions (14/10 h) in the chamber were 26/22/18 ℃ (14/4/6 h), with a relative humidity of 60%. The plants were kept for 15 days under these conditions. After the acclimatization period, half of the plants were watered with a modified Hoagland solution (control) and 60 mM NaCl was added to this solution for the other half. In turn, half of the plants in each irrigation treatment were treated with 0.5 mM salicylic acid (plus 0.01% Tween-20 as a surfactant) every 3 days for 15 days. Furthermore, the effect of a thermal shock was tested by subjecting the plants to a temperature of 43 °C daily for 6 h. The irrigation was increased to maintain 35% drainage, thus avoiding nutrient imbalance in the roots. Thus, there were 8 treatments with 5 repetitions each: plants watered with the control solution, plants watered with the control solution + 60 mM NaCl, plants watered with the control solution + 0.5 mM salicylic acid, and plants watered with the control solution + 60 mM NaCl + 0.5 mM salicylic acid; all these treatments were submitted to ambient temperature (26 ℃) or to heat shock stress (43 ℃).

### 4.2. Plant Growth

Forty plants were analyzed, and the aerial parts were weighed and separated into leaves and stems (including petioles). The water content was calculated from the fresh weights and the dry weights that were determined after a minimum of 72 h at 65 ℃.

### 4.3. Ion Concentrations

Leaf NO_3_^−^ and Cl^−^ were extracted from ground material (0.4 g) with 20 mL of deionized water. They were analyzed in an ion chromatograph (Metrohm 861 Advanced Compact IC; Metrohm 838 Advanced Sampler). The column used was a Metrohm Metrosep A Supp7 250/4.0 mm.

### 4.4. Total Soluble Sugars

The contents of sugars (fructose, sucrose, and glucose) in pepper leaves were measured using an 817 Bioscan ion chromatography system (Metrohm^®^ Ltd., Herisau, Switzerland) equipped with a pulsed amperometric detector (PAD) and a gold electrode. The column used was a METROHM Metrosep Carb 1–150 IC column (4.6 mm × 250 mm), heated to 32 °C.

### 4.5. Polyamines Analysis

The polyamine (PA) contents were studied according to [46], with minor modifications. Leaf samples (2 g) were mixed with 5 mL of perchloric acid (5%), homogenized for 2 min, kept for 1 h under refrigeration with periodic stirring, and then centrifuged at 5000× *g* for 8 min. Each supernatant, containing free PAs, was placed in a plastic jar and kept in a freezer at −20 °C until used. The free PAs were benzoylated by taking 1 mL of each sample and mixing it with 1mL of 2 mol L^−1^ NaOH and 20 μL of benzoyl chloride. The mixture was stirred in a vortex mixer for 15 s and was allowed to rest for 20 min at room temperature. Subsequent to this, 4 mL of a saturated solution of sodium chloride were added, and the system was stirred while 2 mL of diethyl ether were added. The system was left at rest for 30 min, at −20 °C. Then, 1 mL of the diethyl ether phase was taken and evaporated. The residue was resuspended in 0.5 mL of acetonitrile/water (56:44 *v*/*v*). The PAs present were determined with an ACQUITY UPLC system (Waters, Milford, MA, USA) equipped with a UV detector (230 nm) and a reversed-phase column (ACQUITY UPLC HSS T3 1.8 μm, 2.1 mm × 100 mm) maintained at 40 °C. An acetonitrile/water mixture (42:58 *v*/*v*) was used as the elution solvent, with a flow rate of 0.55 mL min^−1^.

### 4.6. Statistical Analysis

The data from the studied parameters were tested for homogeneity of variance and normality of distribution. The SPSS statistical package (IBM SPSS Statistics 25.0, Armonk, NY, USA) was used for the analysis of variance (ANOVA), to determine the effects of temperature, salinity, and salicylic acid, and their interactions. Additionally, Duncan’s multiple range test was used to determine the significance (*p* < 0.05) of the differences among means. Principal component analysis (PCA) was carried out in R, version 4.0.2 [49].

## 5. Conclusions

Exogenous application of SA seems to be a promising management tool to confer heat and salinity stress tolerance. However, when combined, these two stresses elicited a markedly different response in the pepper plants. Logically, the species and variety, the dose and frequency of application, phenological development, the target organ/tissue, and the type of combined stresses considered all the influences of the ability of SA to provide tolerance and thus maintain the global food supply. This study provides new insights into SA-mediated processes; however, future research is of paramount importance to gain a better knowledge of the efficiency and effectiveness as well as the mechanism of action of this hormone in pepper crops under combined stresses.
